# Probing the Single Key Amino Acid Responsible for the Novel Catalytic Function of *ent*-Kaurene Oxidase Supported by NADPH-Cytochrome P450 Reductases in *Tripterygium wilfordii*

**DOI:** 10.3389/fpls.2017.01756

**Published:** 2017-10-13

**Authors:** Ping Su, Hongyu Guan, Yifeng Zhang, Xing Wang, Linhui Gao, Yujun Zhao, Tianyuan Hu, Jiawei Zhou, Baowei Ma, Lichan Tu, Yuru Tong, Luqi Huang, Wei Gao

**Affiliations:** ^1^State Key Laboratory of Dao-di Herbs, National Resource Center for Chinese Materia Medica, China Academy of Chinese Medical Sciences, Beijing, China; ^2^School of Traditional Chinese Medicine, Capital Medical University, Beijing, China; ^3^Beijing University of Chinese Medicine Third Affiliated Hospital, Beijing, China; ^4^Beijing Key Lab of TCM Collateral Disease Theory Research, School of Traditional Chinese Medicine, Capital Medical University, Beijing, China

**Keywords:** *Tripterygium wilfordii*, cytochrome P450 monooxygenase, cytochrome P450 reductase, molecular docking, site-directed mutagenesis, 16α-hydroxy-*ent*-kaurane, 16α-hydroxy-*ent*-kaurenoic acid

## Abstract

*Tripterygium wilfordii* produces not only *ent*-kaurene, which is an intermediate of gibberellin (GA) biosynthesis in flowering plants, but also 16α-hydroxy-*ent*-kaurane, whose physiological role has not been characterized. The two compounds are biosynthesized from the universal diterpenoid precursor (*E*,*E*,*E*)-geranylgeranyl diphosphate (GGPP) by diterpene synthases, which have been discovered and functionally characterized in *T. wilfordii*. Here, we described the functional characterization of four cytochrome P450 reductases (TwCPR) and one *ent*-kaurene oxidase (TwKO). Four *TwCPR*s were found to have relatively ubiquitous expression in *T. wilfordii* root, stem, leaf, and flower tissues. Co-expression of both a TwCPR and TwKO in yeast showed that TwCPR3 has a slightly better activity for providing the electrons required for these reactions, indicating that TwCPR3 is more suitable for use in the functional analysis of other cytochrome P450 monooxygenases. TwKO catalyzed the three-step oxidation of the C4α methyl of the tetracyclic diterpene intermediate *ent*-kaurene to form *ent*-kaurenoic acid as an early step in GA biosynthesis. Notably, TwKO could also convert 16α-hydroxy-*ent*-kaurane to 16α-hydroxy-*ent*-kaurenoic acid, indicating an important function of 16α-hydroxy-*ent*-kaurane in the anti-HIV principle tripterifordin biosynthetic pathway *in planta*. Homology modeling and molecular docking were used to investigate the unknown crucial active amino acid residue involved in the catalytic reaction of TwKO, and one key residue (Leu387) contributed to the formation of 16α-hydroxy-*ent*-kaurenoic acid, most likely by forming hydrogen bonds with the hydroxyl group (-OH) of 16α-hydroxy-*ent*-kaurane, which laid the basis for further investigation of the multifunctional nature of KO catalysis. Also, our findings paved the way for the complete biosynthesis of the anti-HIV principle tripterifordin.

## Introduction

*Tripterygium wilfordii* Hook. f. has a long history of use in traditional Chinese medicine, mainly to treat rheumatoid arthritis (Tu, 2009). Surprisingly, *T. wilfordii* also shows impressive and effective anti-inflammatory, immunosuppressive and antitumor activities (Titov et al., 2011; Chugh et al., 2012; Manzo et al., 2012; Wong et al., 2012; Zhou Z.L. et al., 2012). Much of the remarkable bioactivity can be attributed to bioactive diterpenoids, particularly the abietane-type diterpenoid triptolide. A major challenge to harnessing these natural products is that only small amounts are found *in planta*. Recently, many studies have been targeted toward the investigation of the triptolide biosynthetic pathway in order to directly obtain this bioactive compound using synthetic biology strategies (Andersen-Ranberg et al., 2016; Hansen et al., 2017; Inabuy et al., 2017). However, little attention has been paid to the biosynthesis of the kaurene-type diterpenoids, e.g., bioactive gibberellins (GAs) and anti-HIV principle tripterifordin (Chen et al., 1992).

GAs, which are biosynthesized from the key intermediate *ent*-kaurene through several steps, are a major class of diterpenoid hormones essential to a number of vascular plant growth and developmental processes (Hooley, 1994). To date, the upstream diterpene synthases, also known as *ent*-copalyl diphosphate synthase and *ent*-kaurene synthase, responsible for the formation of *ent*-kaurene has been well investigated in *T. wilfordii* (Andersen-Ranberg et al., 2016; Hansen et al., 2017; Inabuy et al., 2017). Interestingly, the identified *T. wilfordii* diterpene synthases can also catalyze the formation of 16α-hydroxy-*ent*-kaurane, this catalysis was reported in the moss *Physcomitrella patens* and in *Populus trichocarpa* (Hayashi et al., 2006; Irmisch et al., 2015). The downstream enzymes involved in the GA biosynthetic pathway are cytochrome P450 monooxygenases (Yamaguchi, 2008). One of them, *ent*-kaurene oxidase (KO) from the CYP701 family, has been reported to catalyze the three-step oxidation of the C4α methyl of the tetracyclic diterpene intermediate *ent*-kaurene to form *ent*-kaurenoic acid as an early step in GA biosynthesis (Morrone et al., 2010; Hedden and Thomas, 2012). Such catalytic activities strictly depend on their partner, NADPH-cytochrome P450 reductase (CPR), to provide the necessary electrons.

**FIGURE 1:**
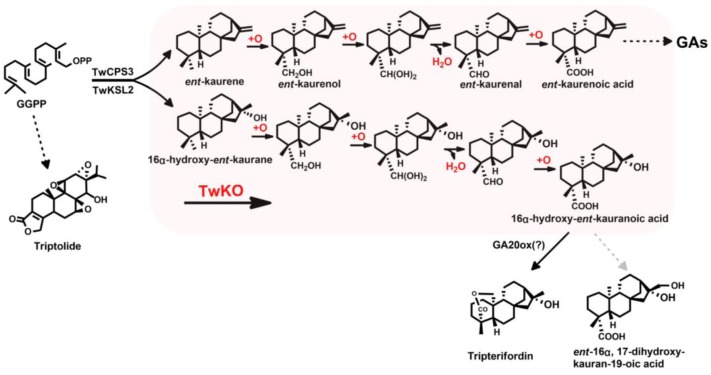
Role of KO in *T. wilfordii* gibberellin phytohormone biosynthesis.

More recently, Mafu et al. (2016) reported the promiscuity of *Oryza sativa* KO (CYP701A6/OsKO2) and *Arabidopsis thaliana* KO (CYP701A3/AtKO). More than 20 labdane-related diterpenoid (LRD) olefins were used to thoroughly investigate the enzymatic promiscuity, and the results showed OsKO2 was much more specific (specific for *ent*-(iso)kaurene and the structurally closely related *ent*-trachylobane), whereas AtKO was found to exhibit much broader promiscuity, targeting the C4α methyl of *ent*-CPP-derived LRDs and the C2α and/or C3β of normal and *syn*-CPP-derived LRDs to produce hydroxylated or carboxylated derivatives (Mafu et al., 2016). These results prompted us to explore the promiscuity of TwKO and to investigate whether it reacted with *ent*-kaurene as well as 16α-hydroxy-*ent*-kaurane in *T. wilfordii*. Herein, we reported the identification of four CPRs and one KO from *T. wilfordii*, and TwKO was confirmed to catalyze *ent*-kaurene to form *ent*-kaurenoic acid, which is consisted with reports in *Salvia miltiorrhiza*, *A. thaliana*, *O. sativa*, *Pisum sativum*, and *P. patens* (Davidson et al., 2004; Sakamoto et al., 2004; Morrone et al., 2010; Miyazaki et al., 2011; Su et al., 2016). Notably, TwKO was discovered to have another new catalytic function, converting 16α-hydroxy-*ent*-kaurane to 16α-hydroxy-*ent*-kaurenoic acid, which most likely is an intermediate of the anti-HIV principle tripterifordin in *T. wilfordii* (**Figure 1**). In addition, the results of Mafu et al. (2016) have driven us to gain insight into the underlying structure–function relationship between this promiscuous enzyme TwKO and its substrates. However, except for GGPPS and terpene synthases (Chang et al., 2006; Mann et al., 2010; Zhou K. et al., 2012), no crystal structure of KO either in plant or in microorganisms has been reported. In the present study, homology modeling and molecular docking were used to construct the three-dimensional structure of TwKO, and one key active amino acid residue (Leu387) was identified to bind to the hydroxyl group (-OH) of 16α-hydroxy-*ent*-kaurane, which laid the basis for further investigation of the multifunctional nature of KO catalysis. Also, the forming of 16α-hydroxy-*ent*-kaurenoic acid catalyzed by TwKO, paved the way for the complete biosynthesis of the tripterifordin and provided the precursor for biosynthesis of other bioactive diterpenoids, e.g., *ent*-16α, 17-dihydroxy-kauran-19-oic acid (Chen et al., 1992; Sung et al., 2011).

## Materials and Methods

### Plant Materials

*Tripterygium wilfordii* suspension cells were cultured in Murashige and Skoog (MS) medium containing 30 g/L sucrose supplemented with 0.1 mg/L kinetin (KT), 0.5 mg/L indole-3-butyric acid (IBA), and 0.5 mg/L 2,4-dichlorophenoxyacetic acid (2,4-D) and maintained at 25 ± 1°C while being shaken at 120 rpm in the dark (Su et al., 2014).

### RNA Isolation, cDNA Synthesis, and Gene Cloning

The total RNA of the induced cells was extracted following the cetyltrimethylammonium bromide (CTAB) method (Del Sal et al., 1989). An aliquot (1 μg) of the total RNA was used to synthesize first-strand cDNA using the PrimeScript 1st Strand cDNA Synthesis Kit in accordance with the manufacturer’s protocol (Takara Bio, Dalian, China).

Mining of the *T. wilfordii* transcriptome (SRA accession number: SRR6001265) was performed, and gene function was annotated based on the following databases: Nr (NCBI non-redundant protein sequences), Nt (NCBI non-redundant nucleotide sequences), Pfam (Protein family), KOG/COG (Clusters of Orthologous Groups of proteins), Swiss-Prot (a manually annotated and reviewed protein sequence database), KEGG Ortholog database, and GO (Gene Ontology) databases. Then, one putative *ent*-kaurene oxidase and four putative CPRs with full-length coding sequences were identified. The full-length cDNAs for the open reading frames (ORFs) of the *TwKO* and *TwCPR* genes were cloned from the cDNA library using PrimeSTAR DNA polymerase (Takara Bio). The PCR products were purified and cloned into a pEASY-Blunt Simple Cloning Vector (TransGen Biotech, Beijing, China), then transformed into *Escherichia coli* Trans5α cells (TransGen Biotech), and cultured in Luria-Bertani (LB) medium at 37°C in the dark. The positive clones were sequenced. All PCR primers used in the experiments are listed in Supplementary Table S1.

### Sequence Analysis

The sequences of the *TwKO*, *TwCPR1*, *TwCPR2*, *TwCPR3*, and *TwCPR4* genes were analyzed using the NCBI database^1^. The ORFs and deduced amino acid sequences were analyzed using the online tool ORF Finder^2^ and the ExPASy online tool^3^, respectively. Multiple sequence alignment was implemented using DNAMAN software. Amino acid sequences for a variety of *ent*-kaurene oxidases were obtained from the NCBI database, and the phylogenetic tree was constructed with MEGA6 software based on the neighbor-joining method (Tamura et al., 2013). One thousand bootstrap replicates were performed during each analysis to define the level of confidence support.

### qRT-PCR Analysis

Total RNA from the root, stem, leaf, and flower of *T. wilfordii* mature plant was extracted following the CTAB method. First-stand cDNA for real-time quantitative PCR was reverse transcribed from total RNA using the FastQuant RT Kit (Tiangen Biotech, Beijing, China). Quantitative reverse transcription PCR (qRT-PCR) were performed with gene specific primers (Supplementary Table S1) and a TransStart Top Green qPCR SuperMix (TransGen Biotech) on a Roche LightCycler 480 Real Time PCR System (Roche, Switzerland). Expression levels were evaluated using the 2^-ΔΔCt^ method (Livak and Schmittgen, 2012) based on the eukaryotic translation elongation factor 1α (Ef1α) as reference gene and with triplicate measurements from five biological replicates.

### Heterologous Expression and Affinity Purification of TwCPRs

To increase protein solubility, four *TwCPR* ORFs were subcloned into the N-terminal MBP fusion expression vector HIS-MBP-pET28a according to the protocol of the *pEASY*^®^-Uni Seamless Cloning and Assembly Kit (TransGen Biotech; HIS-MBP-pET28a was provided by Dr. Xiaohong Zhang. HIS, histidine; MBP, maltose-binding protein. The *MalE* gene encoding MBP was introduced into the *Nde*I and *Bam*HI sites of pET28a). Heterologous expression of four TwCPRs in the *E. coli* strain Transetta (DE3) (TransGen Biotech) was performed as described previously (Su et al., 2017). Briefly, the selected transformants were incubated in 200 mL of LB medium that was supplemented with 50 mg/L kanamycin at 37°C to an OD_600_ of 0.6 ∼ 0.8, after which the temperature was lowered to 16°C for 0.5 h prior to induction with 0.5 mM isopropyl 1-thio-β-D-galactopyranoside (Sigma, United States) for an additional 24 h at 200 rpm. The cell pellets were harvested by centrifugation (3000 *g*, 20 min, 4°C) and stored at -80°C.

The harvested cell pellets were resuspended in 5 mL of binding buffer (20 mM Na_2_HPO_4_, 0.5 M NaCl, 10 mM imidazole, 1 mM DTT, 1 mM PMSF, pH 7.4) and subjected to mild sonication for 20 min (lysed for 10 s, paused for 10 s). The supernatant of the centrifuged lysates (12,000 *g*, 30 min, 4°C) was mixed with Ni-NTA agarose (QIAGEN, WI, United States) at 4°C for 2 h, after which HIS-MBP-tagged purified proteins were acquired following gradient elution with elution buffer (20 mM Na_2_HPO_4_, 0.5 M NaCl, pH 7.4) containing different concentrations of imidazole (50, 100, 250, and 500 mM). The protein concentrations were determined using a Modified Bradford Protein Assay Kit (Sangon Biotech, Shanghai, China).

### Enzyme Assays

Activities of TwCPRs were assayed as described (Yang et al., 2010). All assays and incubations were performed at 25°C in 200 μL of 50 mM Tris buffer (pH 7.4) containing TwCPR enzymes (∼20 μg/mL) and 100 μM cytochrome *c*. The reaction was started by addition of 100 μM NADPH, time-dependent absorbance change at 550 nm was monitored up to 3 min by a Varioskan Flash spectrophotometer (Thermo Fisher Scientific, CA, United States). A molar absorption coefficient of 21/mM/cm for equine heart cytochrome *c* was used for quantification (Guengerich et al., 2009). The reduction of dichlorophenolindophenol (DCPIP, 100 μM) was monitored at 600 nm (20.6/mM/cm); K_3_Fe(CN)_6_ (100 μM) at 424 nm (1.02/mM/cm).

Kinetic parameters for cytochrome *c* reduction were performed as described above with TwCPR enzymes (∼20 μg/mL), 100 μM NADPH and varying cytochrome *c* concentrations (0–200 μM). The kinetic parameters were determined by the non-linear Michaelis–Menten regression equation by using GraphPad Prism 5 (Yang et al., 2010).

### Yeast Expression

Four *TwCPR*s genes were subcloned into the yeast epitope-tagging vector pESC-Leu under the control of the GAL1 inducible promoter using *Bam*HI and *Apa*I sites (Agilent Technologies, United States). Then, the *TwKO* gene was introduced into the four recombinant plasmids above following the protocol of the *pEASY*^®^-Uni Seamless Cloning and Assembly Kit (TransGen Biotech). Each recombinant plasmid containing one of the *TwCPR* genes and the *TwKO* gene was co-expressed in the yeast BY-T20 strain (BY4742, *ΔTrp1*, *Trp1::HIS3-P*_*PGK*1_-*BTS1/ERG20-T*_*ADH*1_-*P*_*TDH*3_-*SaGGPS-T*_*TPI*1_-*P*_*TEF*1_-*tHMG1-T*_*CY C*1_) (Dai et al. 2012, 2013; Shi et al., 2014). To provide the substrate (*ent*-kaurene and 16α-hydroxy-*ent*-kaurane) for TwKO, the substrate-producing module pESC-Trp::TwCPS3/TwKSL2 (whose major product is 16α-hydroxy-*ent*-kaurane) or pESC-Trp::TwCPS3/TwKSL2:A608M (whose major product is *ent*-kaurene) was also transformed into the BY-T20 strains.

The recombinant strains were selected on synthetic dropout medium-Trp-Leu (SD-Trp-Leu) supplemented with 20 g/L glucose and were grown at 30°C for 48–72 h. Single transformed yeast colonies were grown in SD-Trp-Leu liquid medium supplemented with 20 g/L glucose at 30°C during shaking at 230 rpm for 48 h. The yeast cells were pelleted and resuspended in 50 mL of SD-Trp-Leu liquid induction medium supplemented with 20 g/L D-galactose for an additional 48 h. Finally, the induced yeast cells were extracted three times with an equal volume of ethyl acetate. The organic fractions were dried and methylated with (trimethylsilyl)diazomethane (Aladdin Industrial Inc., Shanghai, China) as described previously (Su et al., 2016). The methylated samples were re-dried and then dissolved in 100 μL of ethyl acetate for gas chromatography–mass spectrometry (GC–MS) using a Thermo TRACE 1310/TSQ 8000 gas chromatograph equipped with a TG-5MS (30 m × 0.25 mm × 0.25 μm) capillary column. The GC conditions were as follows: the sample (1 μL) was injected in splitless mode at 250°C under a He flow rate of 1 mL/min in accordance with a temperature program of 1.5 min at 60°C, increased to 200°C at 25°C/min, then to 300°C at 5°C/min. The ion trap heating temperature was 250°C. The electron energy was 70 eV. Spectra were recorded in the range of 40–500 *m/z*.

### Homology Modeling and Molecular Docking

Because the crystal structure of TwKO has not yet been resolved, homology modeling was used to construct the three-dimensional structure of TwKO. After search for templates in the protein database (PDB)^4^, several models were created based on alternative sequence alignments including secondary structure predictions. The crystal structure of cytochrome P450 monooxygenase CYP170A1 from *Streptomyces coelicolor* (PDB ID: 3DBG) was selected as suitable template in view of their high protein sequence similarity and closely related biological functions (Zhao et al., 2009). The academic version of MODELER 9v11 was used for homology modeling of TwKO (Eswar et al., 2003). The structural features in the template protein were used to derive spatial restraints to generate model protein structures using conjugate gradient and simulated annealing optimization procedures (Sali, 1995). After the addition of hydrogen atoms, the energy of the model structures was individually minimized using the staged minimization program of SYBYL X-1.2. First, the simplex method was used for 20 cycles prior to switching to the AMBER FF99 force field for 1000 iterations with the steepest descent calculation. Then, the conjugated gradient calculation was implemented until the convergence on the gradient reached 0.05 kcal/(Å mol). To identify the ligand binding site, the multichannel-surfaces searching method in SYBYL X-1.2 was used to investigate the cavities on the surface of TwKO. The cavity containing active amino acid residues in the catalytic A domain was selected as the active pocket surface to generate a protomol for molecular docking. The protomol was generated using the steric hydrophobic group (CH_4_), the hydrogen bond group (C = O), and the hydrogen acceptor (N–H) within 4.5 Å of the active pocket surface. Surflex-Dock, a well-recognized tool in the field of molecular docking (Wang et al., 2016; Wang X. et al., 2017), was used to calculate the ligand–receptor interaction. 16α-Hydroxy-*ent*-kaurane was prepared as described according to the following procedure: the structure was checked and the hydrogen atoms added, the atomic charges were then added according to the Gasteiger–Hückel method, and energy minimization was implemented using the Tripos force field with 1000 iterations. Then, the optimized compound was docked into the active site of TwKO including the heme in a heteroatom file using the default settings. After each Surflex-Dock run, the 10 best docked conformers or poses were sorted in a molecular spreadsheet; these conformers or poses represented binding affinities in -log 10 (Kd) based on Surflex-Dock scoring function [crash score (also pKd units), polar score, D-score, PMF-score, G-score, ChemSco and CScore] (Spitzer and Jain, 2012). The default parameters of the softwares were used if not stated.

### Site-Directed Mutagenesis of TwKO

Site-directed mutants of TwKO, including the L387A, L387D, L387S, L387T, L387G, and L387R mutants (L, leucine; A, alanine; D, aspartic acid; S, serine; T, threonine; G, glycine; R, arginine), were constructed using the pEASY-Uni Seamless Cloning and Assembly Kit (TransGen Biotech). The mutagenic primer pairs are listed in Supplementary Table S1. The constructed mutants were verified by complete sequencing and then transformed into yeast strains containing the module pESC-Trp::TwCPS3/TwKSL2 and one of the *TwCPR* genes (*TwCPR3*) with higher catalytic activity. The products were extracted and analyzed by GC–MS as described above.

### Statistical Analysis

All assays were performed as at least three independent experiments. The data were presented as the mean ± SD, and statistical comparisons between groups were tested using one-way ANOVA followed by the Dunnett’s test. Differences were considered significant when the *p*-value <0.05.

## Results

### Isolation and Identification of the *TwCPR* Genes

The appropriate CPR is typically required to support the activities of P450. The full-length cDNA sequences of four *TwCPR*s (*TwCPR1*, *TwCPR2*, *TwCPR3*, and *TwCPR4*) were then amplified by RT-PCR, and the total RNA from *T. wilfordii* suspension cells was used as a template.

The amino acid sequence comparison showed that these four CPRs are 84% identical to each other and that all share high sequence identities with known CPRs from other flowering plant species (62-76%) (Mizutani and Ohta, 1998; Guo et al., 2013). The alignment analysis revealed that the four TwCPRs shared many highly conserved amino acid residues and that all of the functional domains were involved in the binding of the P450 monooxygenase. The cofactors of FMN, FAD, and NADPH were also identified (Supplementary Figure S1).

### Kinetics of Recombinant TwCPRs

The recombinant TwCPRs were heterologously expressed in *E. coli*, N-terminally fused to MBP for soluble expression, purified by affinity chromatography (Supplementary Figure S2), and then used for kinetic studies. The results showed that the requirement of the TwCPRs for an electron acceptor is non-specific: cytochrome *c*, K_3_Fe(CN)_6_, and DCPIP could all serve as acceptors (**Table 1**). The kinetic characteristics of the TwCPRs for reducing cytochrome *c* were determined. In general, the four TwCPRs showed similar levels of activity (*K*_cat_ value) (Supplementary Figure S3 and **Table 2**).

**Table 1:** Specific activities of TwCPR1, TwCPR2, TwCPR3, and TwCPR4 in reducing cytochrome *c*, DCPIP, and K_3_Fe(CN)_6_, all at 100 μM, in the presence of 100 μM of NADPH.

	Specific activity (μmol/min/mg protein)		

	Cytochrome *c*	DCPIP	K_3_Fe(CN)_6_
TwCPR1	0.087 ± 0.007	0.056 ± 0.001	0.013 ± 0.006
TwCPR2	0.074 ± 0.007	0.061 ± 0.006	0.038 ± 0.002
TwCPR3	0.121 ± 0.012	0.040 ± 0.003	0.131 ± 0.015
TwCPR4	0.120 ± 0.010	0.048 ± 0.002	0.102 ± 0.001


*Values are presented as mean ± SD.*

**Table 2:** Kinetic constants of recombinant TwCPR1, TwCPR2, TwCPR3, and TwCPR4.

	Cytochrome *c*			

	TwCPR1	TwCPR2	TwCPR3	TwCPR4
*V*_max_ (μmol/min/mg^-1^)	0.246 ± 0.012	0.173 ± 0.005	0.236 ± 0.009	0.229 ± 0.010
*K*_m_ (μM)	12.690 ± 3.848	12.201 ± 1.991	23.413 ± 3.187	23.609 ± 4.095
*K*_cat_ (min^-1^)	31.500	22.137	29.598	28.781
*K*_cat_/*K*_m_	2.482	1.814	1.264	1.219


*Kinetic parameters for cytochrome *c* was carried out with 100 μM NADPH and varying cytochrome *c* concentrations. Values are presented as means ± SD.*

### Expression Analysis of the Identified *TwCPR*s

**FIGURE 2:**
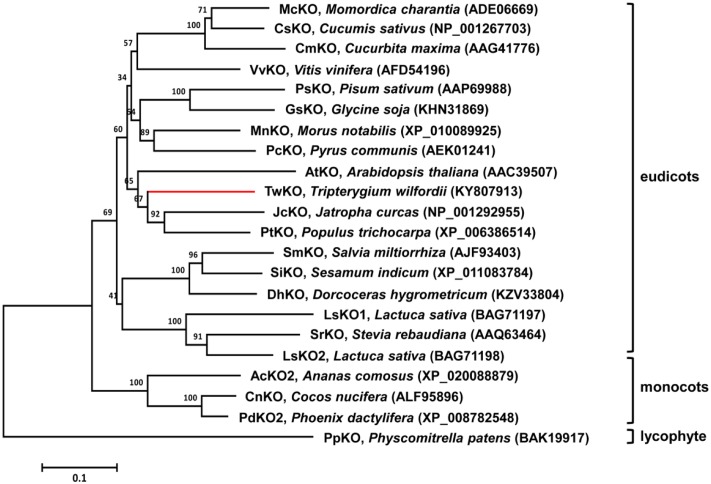
Molecular phylogenetic analysis of selected KOs from different species. The neighbor-joining phylogenetic trees were constructed using the bootstrap method on MEGA6 and the number of bootstrap replications was1000.

To investigate the physiological roles of the identified TwCPRs in *T. wilfordii*, we performed qRT-PCR to evaluate mRNA transcript levels in various organs, including root, stem, leaf, and flower. Four functional *TwCPR*s were found to have relatively ubiquitous expression in all sampled organ tissues. *TwCPR1* showed relative higher transcription levels in all tissues verse other three *TwCPR*s. On the contrary, *TwCPR3* showed the lowest expression in all tissues (**Figure 2**).

### TwCPRs Supported KO Monooxygenase Activity

Previous studies confirmed that TwCPS3 catalyzes the conversion of GGPP to *ent*-CPP. Subsequently, TwKSL2 converts *ent*-CPP to 16α-hydroxy-*ent*-kaurane as a major product via a complex bicyclization and ring rearrangement reaction. Interestingly, changing alanine (A) at the 608th aa of TwKSL2 to methionine (M) leads to the predominant production of *ent*-kaurene, with smaller amounts of 16α-hydroxy-*ent*-kaurane, from *ent*-CPP by the resulting mutant TwKSL2:A608M. Then, we constructed the recombinant plasmids pESC-Trp::TwCPS3/TwKSL2 and pESC-Trp::TwCPS3/TwKSL2:A608M, which we transformed into BY-T20 strains for producing 16α-hydroxy-*ent*-kaurane and *ent*-kaurene, respectively (Supplementary Figure S4).

**FIGURE 3:**
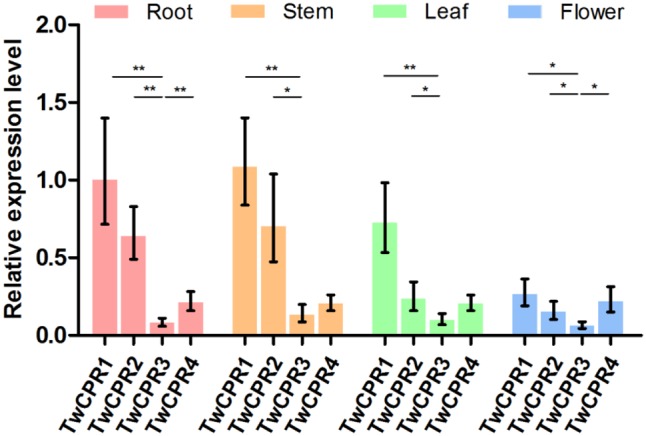
The mRNA expression levels of the *TwCPRs* in root, stem, leaf, and flower tissues from *T. wilfordii*. The expression level was normalized to that of actin. The error bars show the means ± SD value from three technical replicates of at least three biological replicates (^∗^*P* < 0.05, ^∗∗^*P* < 0.01).

TwKO shares 67, 72, and 74% protein sequence identity with AtKO (*A. thaliana*), JcKO (*Jatropha curcas*) and PtKO (*P. trichocarpa*), respectively (**Figure 3**), indicating similar catalytic activity. To characterize the biochemical function of TwKO as well as the TwCPRs *in vivo*, the two complementary enzymes were co-expressed in the constructed *ent*-kaurene- and 16α-hydroxy-*ent*-kaurene-producing yeast strains mentioned above. After the extraction and methylation of the fermentation products, *ent*-kaurenoic acid methyl ester (*ent*-kaurenoic acid-Me) was detected by comparison with the authentic methylated standard (Sigma, United States). Another product with a major molecular ion peak at *m/z* 334 [M]^+^ was also identified as 16α-hydroxy-*ent*-kaurenoic acid methyl ester (16α-hydroxy-*ent*-kaurenoic acid-Me) by comparison with data from the NIST mass spectral database.

**FIGURE 4:**
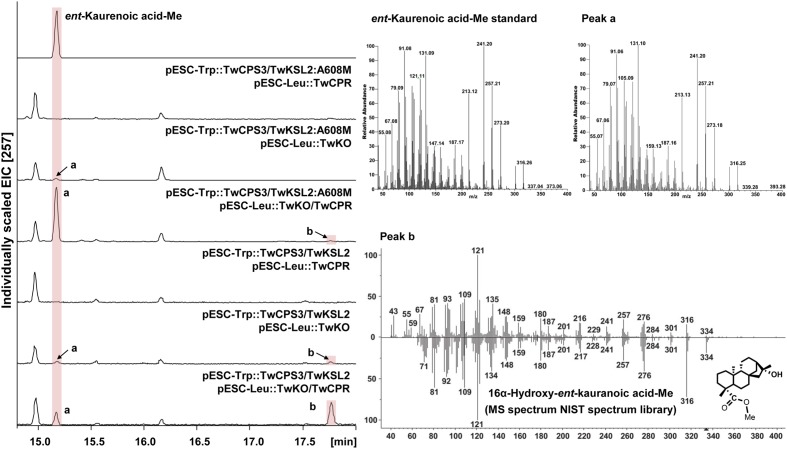
GC–MS analysis of *in vivo* assay for TwKO activity. Peak a and b, methyl ester derivatives of the products from yeast strain BY-T20 cultures expressing TwKO combined with TwCPR, TwCPS3, and TwKSL2 (or TwKSL2:A608M), and their corresponding mass spectrums (Peak a, *R_t_* = 15.17 min; Peak b, *R_t_* = 17.77 min). Mass spectrum of peak a was identical to that of the authentic standard, and peak b was identified by comparison with the NIST spectrum library.

**FIGURE 5:**
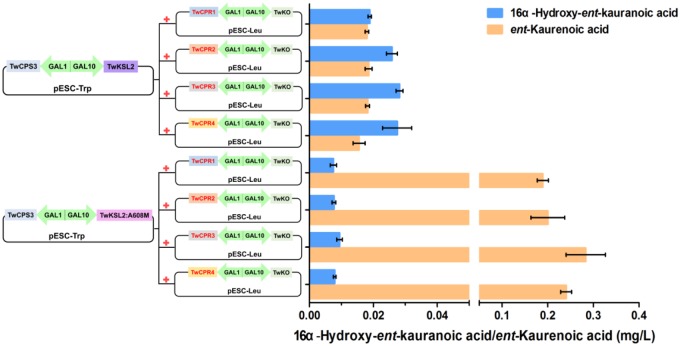
16α-Hydroxy-*ent*-kaurenoic acid/*ent*-kaurenoic acid production by recombinant yeasts harboring modules overexpressing various enzymes. The strains were cultivated for 48 h in synthetic dropin media, and the products were extracted with equal volume of ethyl acetate and methylated with (trimethylsilyl)diazomethane. Values shown are means ± SD of at least three biological replicates.

Co-expression of TwKO and the TwCPRs, in conjunction with the recombinant plasmid pESC-Trp::TwCPS3/TwKSL2:A608M, led to the predominant product *ent*-kaurenoic acid-Me, as well as smaller amounts of 16α-hydroxy-*ent*-kaurenoic acid-Me; while roughly equal amounts of 16α-hydroxy-*ent*-kaurenoic acid-Me and *ent*-kaurenoic acid-Me were produced, along with the recombinant plasmid pESC-Trp::TwCPS3/TwKSL2 (**Figure 4**). These results confirmed that *TwKO* encodes a functional *ent*-kaurene oxidase that is involved not only in the three-stage oxidation of *ent*-kaurene to *ent*-kaurenoic acid in the *T. wilfordii* GA biosynthetic pathway but also in the continuously oxidation of 16α-hydroxy-*ent*-kaurane to 16α-hydroxy-*ent*-kaurenoic acid in an unreported diterpenoid biosynthetic pathway. Different combinations of each TwCPR and TwKO produced distinct levels of 16α-hydroxy-*ent*-kaurenoic acid/*ent*-kaurenoic acid, and the combination of TwCPR3 and TwKO generated the highest content of 16α-hydroxy-*ent*-kaurenoic acid as well as *ent*-kaurenoic acid, indicating that TwCPR3 has slightly better activity for providing the electrons required for these reactions (**Figure 5**).

### Homology Modeling and Molecular Docking

TwKO has shown promiscuous enzymatic catalysis. This promiscuity was very recently reported for AtKO in *A. thaliana* (Mafu et al., 2016) and prompted us to screen the crucial active amino acid binding of a key group of substrates, providing insight into the catalytic mechanism. However, no crystal structure of KO in plants or in microorganisms has been reported. The crystal structure of cytochrome P450 CYP170A1 from *S. coelicolor* (Zhao et al., 2009) was selected as template structure used for homology modeling and molecular docking, and the values of max score, total score, query cover, *E*-value, and ident were 81.6, 81.6, 39%, 4e^-16^, and 29% for TwKO. We chose this structure as a template for establishing the 3D structures of TwKO. Modeller 4.0 software was used to generate the sequence alignment of the template protein with TwKO (Kuntal et al., 2010).

**FIGURE 6:**
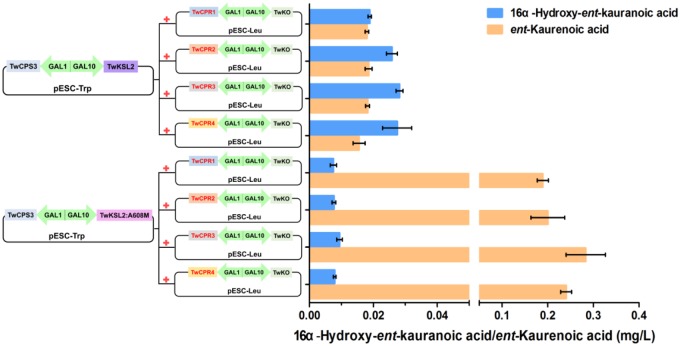
Binding modes of TwKO with 16α-hydroxy-*ent*-kaurane (yellow) and heme (green carbon atoms). The models were generated using 3DBG (structure of human *Streptomyces coelicolor* cytochrome P450 monooxygenase CYP170A1) as a template. The hydrogen bonding interactions are displayed in dotted lines.

According to the multichannel-surfaces module, five cavities were produced from on the surface of TwKO. Previous studies suggested that the catalytic A domain of KO is associated with substrate binding and an oxygen pocket (Supplementary Figure S5) (Davidson et al., 2004). In this study, the corresponding location in TwKO, which covers most of the reported key amino acid residues, was selected as the active site to generate the protomol for molecule docking. 16α-Hydroxy-*ent*-kaurane was docked into the active site of TwKO using the Surflex-Dock program of the SYBYL X-1.2 software package. The binding mode of this compound with TwKO was predicted (**Figure 6**). So far, the docking can only detect the hydrogen-bond interaction. Only Leu387 interacted with the compound and showed a hydrogen-bond interaction with the compound, implying that Leu387 of TwKO is the key amino acid residue binding to this compound.

### Site-Directed Mutagenesis of the TwKO Proteins

**FIGURE 7:**
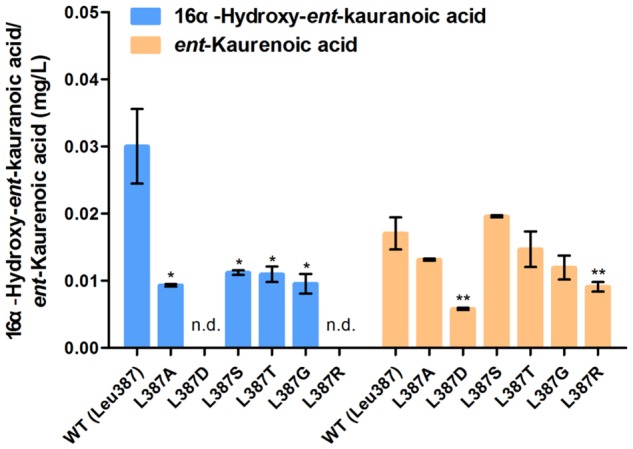
Relative catalytic activity of TwKO mutants when *ent*-kaurene and 16α-hydroxy-*ent*-kaurane were used as substrates. Means ± SD of three biological replicates are shown (^∗^*P* < 0.05, ^∗∗^*P* < 0.01).

We predicted key catalytic sites by molecular docking. Structural comparisons suggested that a crucial amino acid, Leu387 of TwKO, may form hydrogen bonds with the hydroxyl group (-OH) of 16α-hydroxy-*ent*-kaurane, which is involved in continuous oxidation reactions. To preliminarily identify the biochemical impact of this key amino acid, we mutated Leu387 to six other amino acids that have different properties. Site-directed mutagenesis and enzyme assays demonstrated that the L387A, L387D, L387T, L387G, and L387R mutations led to an approximate 13.7–65.9% decrease; the L387S mutation led to an approximate 15.0% increase in TwKO activity toward *ent*-kaurenoic acid; and the L387A, L387S, L387T, and L387G mutations resulted in an approximate 62.7–69.0% decrease in TwKO activity toward 16α-hydroxy-*ent*-kaurenoic acid. Notably, both the L387D and L387R mutations caused the loss of the specific catalytic function from 16α-hydroxy-*ent*-kaurane to 16α-hydroxy-*ent*-kaurenoic acid (**Figure 7**).

## Discussion

Since the discovery of the notable anti-inflammatory, immunosuppressive and antitumor activity of diterpenoids, particularly the abietane-type diterpenoid triptolide, multiple studies have been reported to target toward the investigation of the triptolide biosynthetic pathway in order to directly obtain this bioactive compound using synthetic biology strategies (Andersen-Ranberg et al., 2016; Hansen et al., 2017; Inabuy et al., 2017). However, little attention has been paid to the biosynthesis of the kaurene-type diterpenoids, e.g., bioactive GAs and anti-HIV principle tripterifordin. Here, we developed the *T. wilfordii* transcriptome library and searched in particular for enzymes involved in the kaurene-type diterpenoids biosynthesis. Then, we identified an *ent*-kaurene oxidase (TwKO), as well as four NADPH-CPRs (TwCPRs) in *T. wilfordii*.

### Functional Characterization and Tissue-Specific Expression Analysis of the *TwCPR*s

In plants, thousands of metabolites are generated in response to environmental change, and P450s play vital roles in the biosynthesis of these metabolites. To support enzyme activities of different P450s, higher plants possess more than one *CPR*. These *CPR* genes include at least one constitutively expressed *CPR* to support their growth and development and other inducible expressed *CPR*s to cope with environmental challenges (Nicotra et al., 2010). In the work presented here, four *TwCPR* genes were isolated and characterized for their catalytic properties, and tissue-specific expression analysis.

To our knowledge, the amino acid sequences of CPRs in plants shared relatively high homology, but their potential as electron donors for P450 monooxygenases seem to be different. Guo et al. (2013) found that *S. miltiorrhiza* CPR1 (SmCPR1) was more competent than SmCPR2 to support the activity of the SmCYP76AH1 for producing ferruginol at a titer of 10.5 and 5.2 mg/L in the engineered yeasts. Although four TwCPRs showed similar levels of activity (Supplementary Figure S3 and **Table 2**), co-expression with TwKO in yeast showed TwCPR3 has slightly better activity for providing the electrons to support TwKO activity with D-galactose treatment (**Figure 5**), indicating that TwCPR3 was most likely to be the best choose for use in the functional analysis of other cytochrome P450 monooxygenases. *TwCPR*s were found to have relatively ubiquitous expression in all sampled organ tissues (root, stem, leaf, and flower). *TwCPR1* showed relative higher transcription levels in all tissues verse other three *TwCPR*s, suggesting that *TwCPR1* was predominant electron donors responsible for metabolites biosynthesis *in planta* (**Figure 2**). *TwCPR3* showed the lowest expression in all tissues (root, stem, leaf, and flower), implying that *TwCPR3* might be an inducible expressed *CPR* responded to exogenous environmental stimuli (e.g., D-galactose) similar to the reported *Andrographis paniculata CPR*2 gene (Lin et al., 2017).

### Novel Catalytic Function of TwKO

Bioactive GAs are diterpene plant hormones that are biosynthesized through complex pathways and control diverse aspects of growth and development (Yamaguchi, 2008; Araújo and Fernie, 2012; Schwechheimer, 2012). *T. wilfordii* produces not only *ent*-kaurene as a key intermediate to GAs, but also 16α-hydroxy-*ent*-kaurane, whose physiological role is unknown. Functional characterization of TwKO revealed that it could catalyze three-step oxidation of the C4α methyl of the tetracyclic diterpene intermediate *ent*-kaurene to form *ent*-kaurenoic acid as an early step in GA biosynthesis. Notably, it could also convert 16α-hydroxy-*ent*-kaurane to 16α-hydroxy-*ent*-kaurenoic acid. In *P. patens*, 16α-hydroxy-*ent*-kaurane has no physiological role in growth and development and is directly released into the air (Von Schwartzenberg et al., 2004), while the role of this hydroxylated compound in *P. trichocarpa* could be the precursor for other yet unidentified compounds and function as an allelochemical or phytoanticipin (Irmisch et al., 2015).

In *T. wilfordii*, tripterifordin was reported to inhibit HIV replication in H9 lymphocyte cells with an EC_50_ of 1 μg/mL (6 μM), while its biosynthetic pathway is unknown. Here, we confirmed that 16α-hydroxy-*ent*-kaurane was transformed into 16α-hydroxy-*ent*-kaurenoic acid by TwKO, then could be converted to anti-HIV principle tripterifordin catalyzed by an unreported gibberellin 20-oxidase (GA20ox) (Phillips et al., 1995; Kawai et al., 2014). It is the first time to clarify the physiological role of 16α-hydroxy-*ent*-kaurane, which is the precursor for tripterifordin biosynthesis in *T. wilfordii*. Another bioactive compound *ent*-16α, 17-dihydroxy-kauran-19-oic acid in *Siegesbeckia pubescens* could regenerate epidermal tissue mainly through epithelial growth factor receptor phosphorylation, indicating this compound might be applied to wound-healing agents and to a basic materials used in cosmetics (Sung et al., 2011). 16α-Hydroxy-*ent*-kaurane can be also used as an intermediate to synthesize the bioactive *ent*-16α, 17-dihydroxy-kauran-19-oic acid, via one-step oxidation of the C17 methyl of 16α-hydroxy-*ent*-kaurenoic acid (**Figure 1**). In short, these findings paved the way for the complete biosynthesis of the tripterifordin and provided the precursor for biosynthesis of other bioactive diterpenoids.

### Assumed Catalytic Mechanism of TwKO

In recent years, many reports have indicated that the substitution of a single amino acid in *ent*-kaurene synthase and cytochrome P450 oxygenases may change the reaction products (Xu et al., 2007; Jia and Peters, 2016; Scheler et al., 2016), driving us to find out the crucial residue related to the catalytic function of TwKO. The crystal structure of *S. coelicolor* CYP170A1 was used for homology modeling to construct the three-dimensional structure of TwKO (Zhao et al., 2009), and one key residue (Leu387) was identified to form hydrogen bonds with the hydroxy group (-OH) of 16α-hydroxy-*ent*-kaurane (**Figure 6**). All of the six mutants (L387A, L387D, L387S, L387T, L387G, and L387R) effect the TwKO activity toward *ent*-kaurenoic acid and 16α-hydroxy-*ent*-kaurenoic acid. In particular, L387D and L387R mutants led to an approximate 65.9 and 46.7% decrease in TwKO activity toward *ent*-kaurenoic acid, and caused the loss of the specific catalytic function from 16α-hydroxy-*ent*-kaurane to 16α-hydroxy-*ent*-kaurenoic acid (**Figure 7**).

These results suggested that Leu387 interacted with the hydroxy group (-OH) of 16α-hydroxy-*ent*-kaurane to form hydrogen bond, which contributed to form a catalytic conformation with high catalytic activity toward 16α-hydroxy-*ent*-kaurenoic acid. When the residue Leu387 was mutated to other amino acids, the space structure of protein residue would be affected and the hydrophilicity/hydrophobicity of the side chain might change, leading to the change of the distance of hydrogen bonding or hydrogen bonding sites. All these changes likely alter the catalytic conformation, which increases or reduces the entropy costs of substrate binding to the catalytic site and thus reduces or increases catalytic activity (Wang J. et al., 2017). Taking the L387D mutant as an example, the hydrophobic side chain of Leu was changed to the hydrophilic side chain of Asp, leading to the hydroxyl group of 16α-hydroxy-*ent*-kaurane interacted with the main chain of Arg388 to form H-bond (Supplementary Figure S6A). And this change maybe change the catalytic conformation and greatly increase the entropy costs of substrate binding to the catalytic site and thus reduce catalytic activity. As to the L387D and L387S mutants, the residue side chains were altered, resulting in hydrogen bonds could not be formed because of the increased steric hindrance in the bonding position (Supplementary Figures S6B,C). The entropy costs of substrate binding to the catalytic site were increased, thus reducing catalytic activity. Because *ent*-kaurene does not contain a group providing hydrogen-bonding interaction, the molecular docking results did not show hydrogen bonding interactions between TwKO with *ent*-kaurene, providing little useful information to explain the how these mutants affect the specific catalytic function from *ent*-kaurene to *ent*-kaurenoic acid. To confirm the assumed catalytic mechanism of TwKO calls for further study.

## Author Contributions

PS, LH, and WG conceived and designed the research. PS, HG, YfZ, and LG performed the experiments. XW performed the homology modeling and molecular docking. YjZ, TH, JZ, BM, LT, and YT participated in the research and analyzed the data. PS, WG, and LH wrote the paper with contributions of all the authors.

## Conflict of Interest Statement

The authors declare that the research was conducted in the absence of any commercial or financial relationships that could be construed as a potential conflict of interest.

## Footnotes

1 http://www.ncbi.nlm.nih.gov/

2 http://www.ncbi.nlm.nih.gov/gorf/gorf.html

3 http://web.expasy.org/translate/

4 http://www.rcsb.org/pdb/

## Abbreviations

CPR: cytochrome P450 reductase
CTAB: cetyltrimethylammonium bromide
2,4-D: 2,4-dichlorophenoxyacetic acid
DCPIP: dichlorophenolindophenol
*ent*-CPP: *ent*-copalyl diphosphate
GAs: gibberellins
GC–MS: gas chromatography–mass spectrometer
GGPP: geranylgeranyl diphosphate
GO: Gene Ontology database
IBA: indole-3-butyric acid
KO: *ent*-kaurene oxidase
KOG/COG: Clusters of Orthologous Groups of proteins
KT: kinetin
LB: Luria-Bertani
LRD: labdane-related diterpenoid
MBP: maltose-binding protein
MS: Murashige and Skoog
Nr: NCBI non-redundant protein sequences
Nt: NCBI non-redundant nucleotide sequences
ORF: open reading frame
Pfam: Protein family
Swiss-Prot: a manually annotated and reviewed protein sequence database
