# Supplementary material for: Probing the Single Key Amino Acid Responsible for the Novel Catalytic Function of ent-Kaurene Oxidase Supported by NADPH-Cytochrome P450 Reductases in Tripterygium wilfordii
Source: Front Plant Sci. 2017 Oct 13;8:1756. doi: 10.3389/fpls.2017.01756 (PMC5645531; doi:10.3389/fpls.2017.01756)
Supplement: Supplementary file 1 [file Data_Sheet_1.doc]

**Probing the Single Key Amino Acid Responsible for the Novel Catalytic Function of *ent*-Kaurene Oxidase Supported by NADPH-Cytochrome P450 Reductases in *Tripterygium wilfordii***

Running title: *ent*-Kaurene oxidase in *Tripterygium wilfordii*.

Ping Su^1,2^, Hongyu Guan^1,2,3^, Yifeng Zhang^1,2^, Xing Wang^2,4^, Linhui Gao^1,2^, Yujun Zhao^1^, Tianyuan Hu^2^, Jiawei Zhou^2^, Baowei Ma^2^, Lichan Tu^2^, Yuru Tong^1,2^, Luqi Huang^1*^, and Wei Gao^2,4*^

^1^State Key Laboratory of Dao-di Herbs, National Resource Center for Chinese Materia Medica, Chinese Academy of Chinese Medical Sciences, Beijing, China

^2^School of Traditional Chinese Medicine, Capital Medical University, Beijing China

^3^Beijing University of Chinese Medicine Third Affiliated Hospital, Beijing China

^4^Beijing Key Lab of TCM Collateral Disease Theory Research, School of Traditional Chinese Medicine, Capital Medical University, Beijing China

Correspondence:

Luqi Huang

Tel.: 86-10-84044340

Fax: 86-10-84027175

huangluqi01@126.com

Wei Gao

86-10-8391-6572

Fax: 86-10-8391-1627

[weigao@ccmu.edu.cn](mailto:weigao@ccmu.edu.cn)

**Supplementary Table S1. Primers used in this study.**

| Primer Name | Primer sequence (5`→3`) |
| --- | --- |
| **ORF-PCR** | |
| TwKO-F | ATGGCGGCTCCAACTCCTTCT |
| TwKO-R | TCAATTTCTTGGCTTTATCATTGCG |
| TwCPR1-F | ATGCAATCTTCTTCGGATTCTAT |
| TwCPR1-R | TTACCATACATCCCGGAGATACCTTC |
| TwCPR2-F | ATGCAATCTTCTTCGGATTCTAT |
| TwCPR2-R | TTACCATACATCCCGGAGATACCTTC |
| TwCPR3-F | ATGAGTTCGAGCTCGGATTTGG |
| TwCPR3-R | TCACCAAACATCTCGAAGATATCTTC |
| TwCPR4-F | ATGAGTTCGAGCTCGGATTTGG |
| TwCPR4-R | TCACCAAACATCTCGAAGATATCTTC |
| **ORF-PCR (HIS-MBP-pET28a)** | |
| TwCPR1-F | GTTCCAGGGGCCCGAATTCGGAATGCAATCTTCTTCGGATTCTAT |
| TwCPR1-R | GTCGACGGAGCTCGAATTCGGCCATACATCCCGGAGATACCTTC |
| TwCPR2-F | GTTCCAGGGGCCCGAATTCGGAATGCAATCTTCTTCGGATTCTAT |
| TwCPR2-R | GTCGACGGAGCTCGAATTCGGCCATACATCCCGGAGATACCTTC |
| TwCPR3-F | GTTCCAGGGGCCCGAATTCGGAATGAGTTCGAGCTCGGATTTGG |
| TwCPR3-R | GTCGACGGAGCTCGAATTCGGCCAAACATCTCGAAGATATCTTC |
| TwCPR4-F | GTTCCAGGGGCCCGAATTCGGAATGAGTTCGAGCTCGGATTTGG |
| TwCPR4-R | GTCGACGGAGCTCGAATTCGGCCAAACATCTCGAAGATATCTTC |
| **ORF-PCR (pESC-Leu)** | |
| TwCPR1-*BamH*I-F | CGCGGATCCGATGCAATCTTCTTCGGATTCTAT |
| TwCPR1-*Apa*I-R | TCGGGGCCCAACCATACATCCCGGAGATACCTTC |
| TwCPR2-*BamH*I-F | CGCGGATCCGATGCAATCTTCTTCGGATTCTAT |
| TwCPR2-*Apa*I-R | TCGGGGCCCAACCATACATCCCGGAGATACCTTC |
| TwCPR3-*BamH*I-F | CGCGGATCCGATGAGTTCGAGCTCGGATTTGG |
| TwCPR3-*Apa*I-R | TCGGGGCCCAACCAAACATCTCGAAGATATCTTC |
| TwCPR4-*BamH*I-F | CGCGGATCCGATGAGTTCGAGCTCGGATTTGG |
| TwCPR4-*Apa*I-R | TCGGGGCCCAACCAAACATCTCGAAGATATCTTC |
| TwKO-F | ACCCTCACTAAAGGGCGATGGCGGCTCCAACTCCTTCTT |
| TwKO-R | TCCATCGATACTAGTGCATTTCTTGGCTTTATCATTGCG |
| **Site-directed mutation (pESC-Leu)** | |
| TwKO-F1 | ACCCTCACTAAAGGGCGATGGCGGCTCCAACTCCTTCTT |
| TwKO-R2 | TCCATCGATACTAGTGCATTTCTTGGCTTTATCATTGCG |
| TwKOL387A-F2 | GGCTCCTGTGGTCCCTGCACGATATGCACATGAAGATAC |
| TwKOL387A-R1 | GTATCTTCATGTGCATATCGTGCAGGGACCACAGGAGCC |
| TwKOL387R-F2 | GGCTCCTGTGGTCCCTCGACGATATGCACATGAAGATAC |
| TwKOL387R-R1 | GTATCTTCATGTGCATATCGTCGAGGGACCACAGGAGCC |
| TwKOL387D-F2 | GGCTCCTGTGGTCCCTGACCGATATGCACATGAAGATAC |
| TwKOL387D-R1 | GTATCTTCATGTGCATATCGGTCAGGGACCACAGGAGCC |
| TwKOL387G-F2 | GGCTCCTGTGGTCCCTGGACGATATGCACATGAAGATAC |
| TwKOL387G-R1 | GTATCTTCATGTGCATATCGTCCAGGGACCACAGGAGCC |
| TwKOL387S-F2 | GGCTCCTGTGGTCCCTTCACGATATGCACATGAAGATAC |
| TwKOL387S-R1 | GTATCTTCATGTGCATATCGTGAAGGGACCACAGGAGCC |
| TwKOL387T-F2 | GGCTCCTGTGGTCCCTACACGATATGCACATGAAGATAC |
| TwKOL387T-R1 | GTATCTTCATGTGCATATCGTGTAGGGACCACAGGAGCC |
| **qRT-PCR** | |
| TwCPR1-F | ACGGAAACTGTTGAGGAAGCAGTTA |
| TwCPR1-R | GTTGGGTCAGAAGCATGGGC |
| TwCPR2-F | AATTTATCAGAAACGGTTGAGGAGG |
| TwCPR2-R | GCATAGCGAGCAAGAGCCGT |
| TwCPR3-F | ATGTTGGGGTGTTTGCGG |
| TwCPR3-R | CAAAGCAATCAACGCAGCCT |
| TwCPR4-F | CTGGAGACCATGTCGGTGTGTA |
| TwCPR4-R | GCAGCCTTTTTAGGAGCATTAAG |
| Ef1α-F | CCAAGGGTGAAAGCAAGGAGAGC |
| Ef1α-R | CACTGGTGGTTTTGAGGCTGGTATCT |

* The underlined bases represent the restriction sites used for cloning. F, forward; R, reverse.


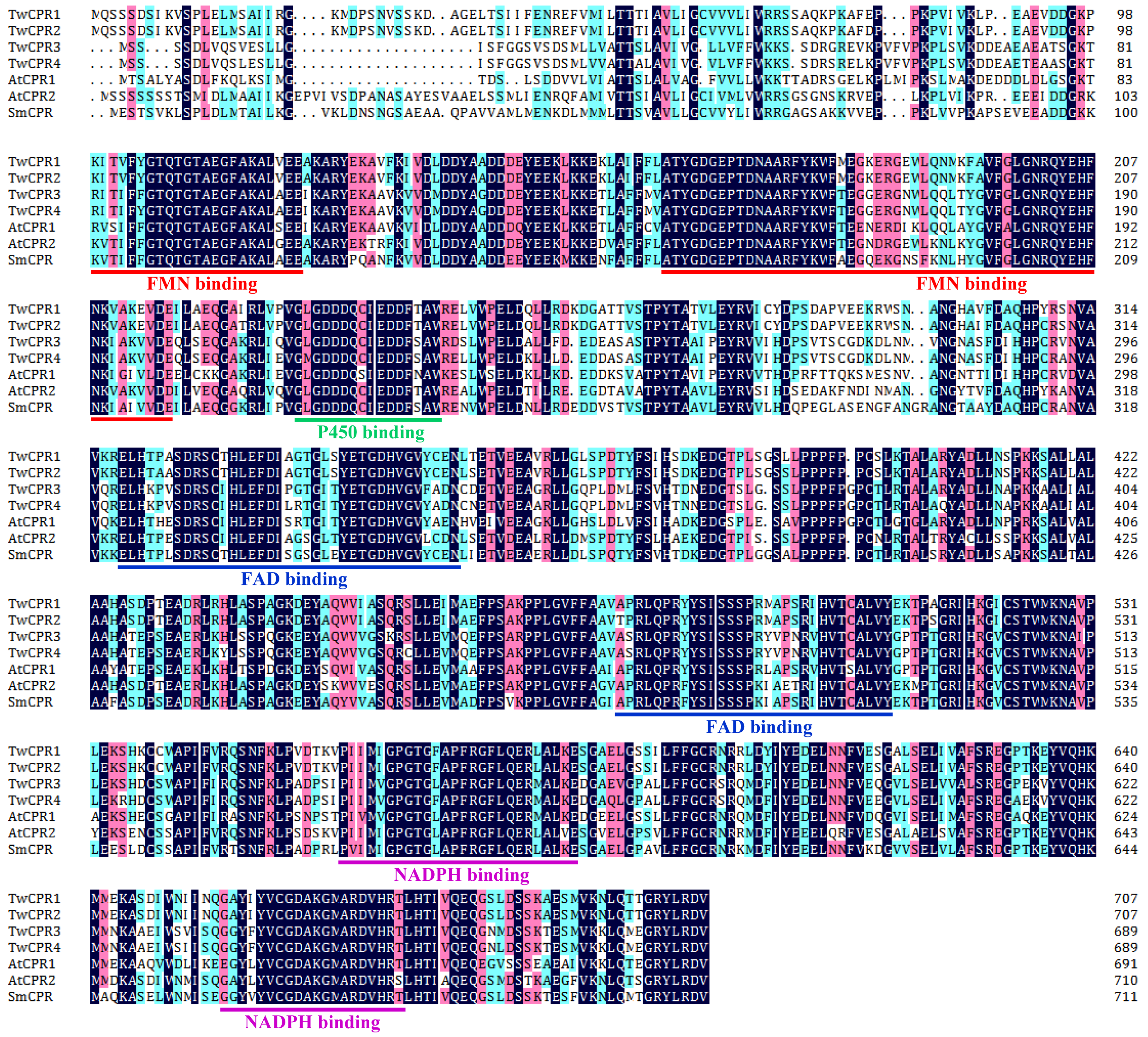


**Supplementary Figure S1. Multiple sequence alignment of deduced amino acid sequences of TwCPRs with other CPR homologs.** CPRs used for alignment were from *Tripterygium wilfordii* (TwCPR1: MF135233, TwCPR2: MF135234, TwCPR3: MF135235, TwCPR4: MF135236), *Arabidopsis thaliana* (AtCPR1: NP_194183, AtCPR2: NP_194750), and *Salvia miltiorrhiza* (SmCPR: AGL46979); Putative conserved FMN-, cytochrome P450-, FAD- and NADPH-binding regions are underlined.

**
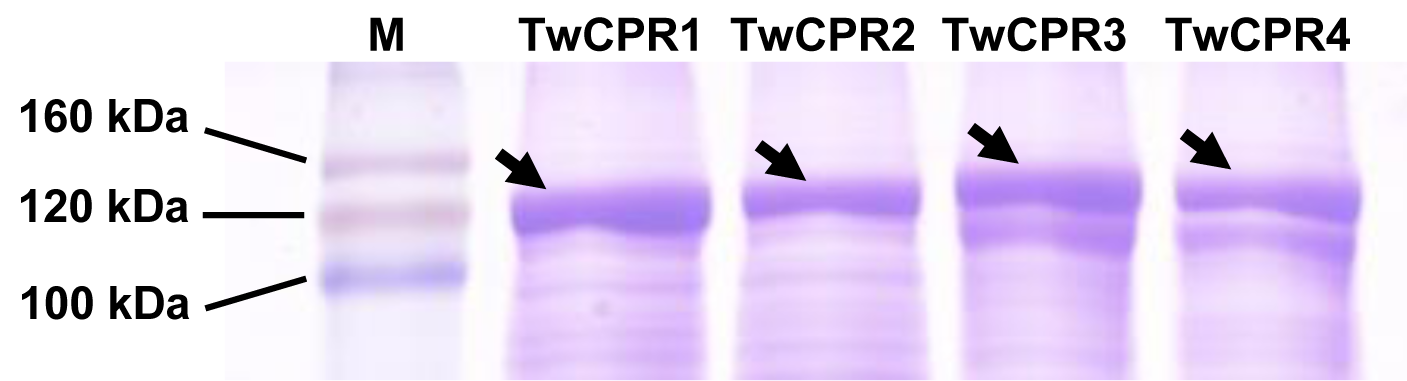
**

**Supplementary Figure S2.** **Sodium dodecyl sulfatepolyacrylamide gel electrophoresis (SDS-PAGE) analysis of HIS-MBP-tagged TwCPR1, TwCPR2, TwCPR3 and TwCPR4 through affinity purification with** **Ni-NTA agarose.** The arrows indicate the purified recombinant proteins. **M**, standard protein markers (TransGen Biotech).


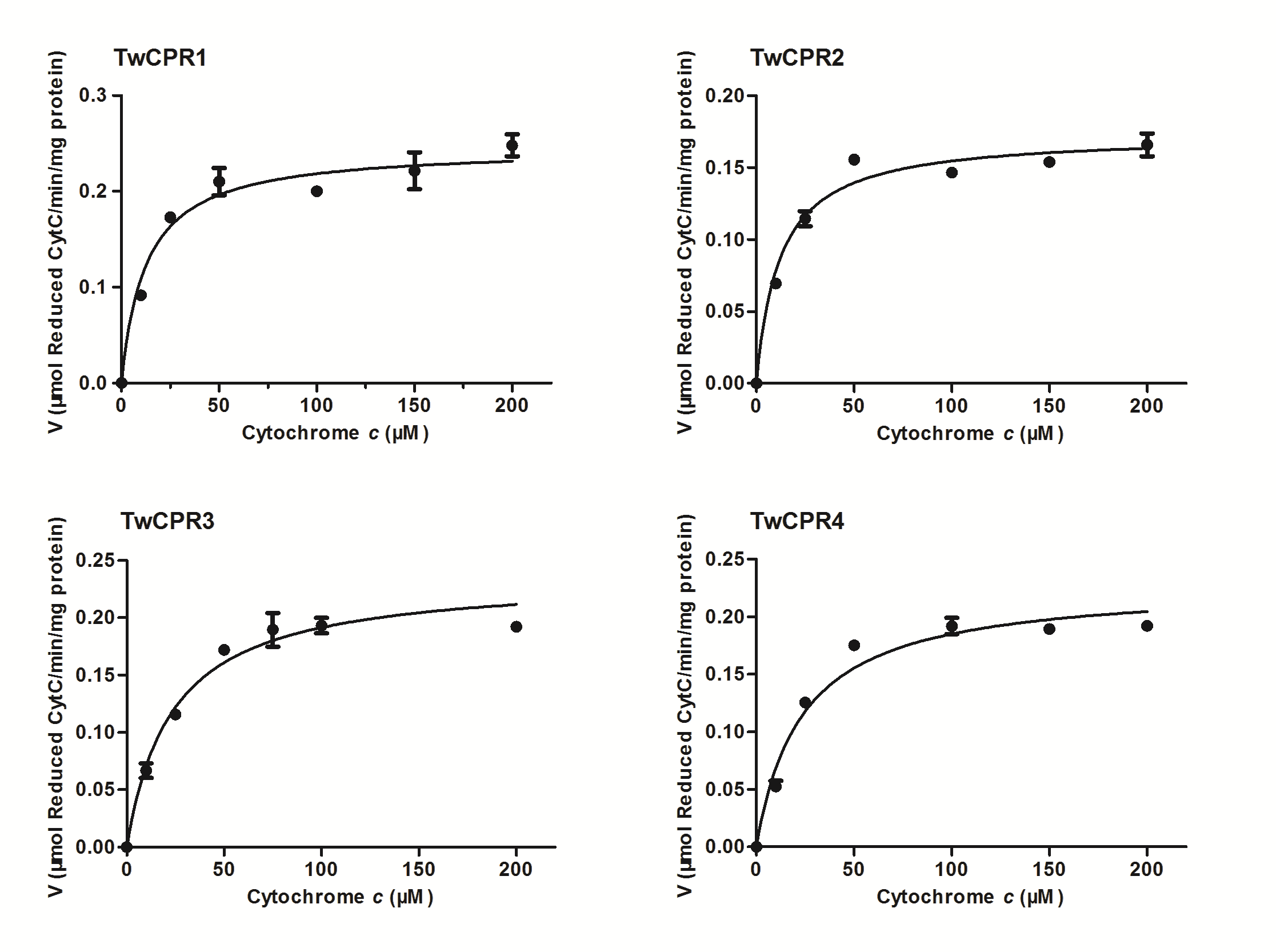


**Supplementary Figure S3. Kinetic analysis of the recombinant TwCPRs.** Substrate saturation of CPR with increasing cytochrome *c* concentrations at 100 μM NADPH. Velocities are expressed as micromole reduced cytochrome *c* produced per minute per microgram of proteins. Data was fitted using GraphPad Prism 5 software.


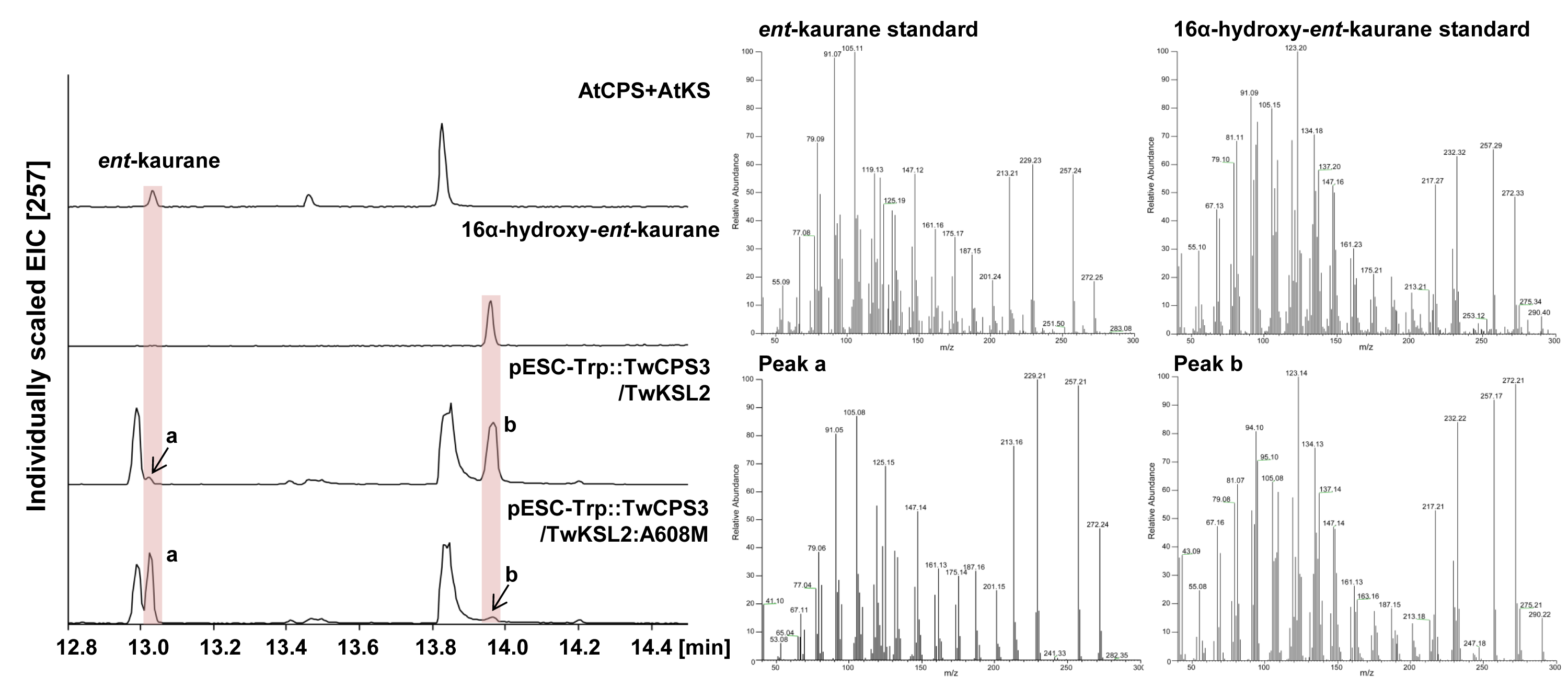


**Supplementary Figure S4. GC–MS analysis of the products of *ent*-kaurene & 16α-hydroxy-*ent*-kaurane producing yeast strains.** **AtCPS+AtKS:** the formation of *ent*-kaurene *in vivo* assay catalyzed by AtCPS+AtKS from GGPP. **Peak a and b**, the products from yeast strain BY-T20 cultures expressing TwCPS3 and TwKSL2 (or TwKSL2:A608M), and their corresponding mass spectrums (**Peak a**, *R_t_* = 13.02 min; **Peak b**, *R_t_* = 13.96 min).


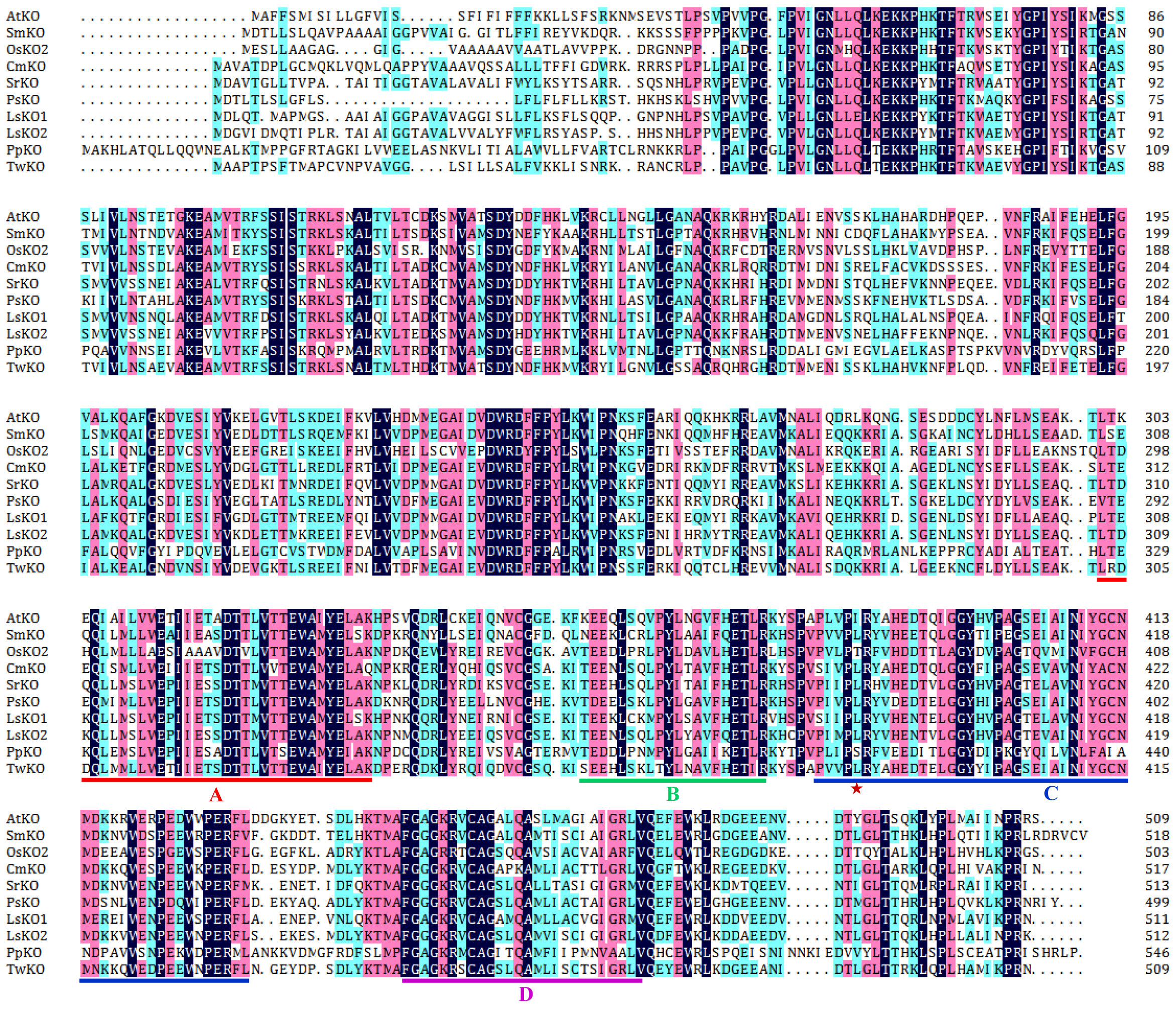


**Supplementary Figure S5.** **Multiple sequence alignment of deduced amino acid sequences of TwKO with other KO homologs.** KO used for alignment were from *A. thaliana* (AtKO: AAC39507), *S. miltiorrhiza* (SmKO: AJF93403), *Oryza sativa* (OsKO2: AAT81229), *Cucurbita maxima* (CmKO: AAG41776), *Stevia rebaudiana* (SrKO: AAQ63464), *Pisum sativum* (PsKO: AAP69988), *Lactuca sativa* (LsKO1: BAG71197), *L. sativa* (LsKO2: BAG71198), *Physcomitrella patens* (PpKO: BAK19917), and *T. wilfordii* (TwKO, KY807913). The red asterisk represents the position of Leu387 in TwKO. The catalytic domains A to D are underlined. The catalytic A domain is associated with substrate binding and oxygen pocket and traverses the distal surface of the haem. The B domain contains the EXXR section of the highly conserved E,R,R triad involved in positioning the haem-binding pocket. The C domain has the PERF clan designator region that includes the third R of the E,R,R triad. The D domain has the haem-binding region associated with all cytochrome P450s with the Cys that provides the axial thiolate ligand to the haem iron (Davidson et al., 2004).


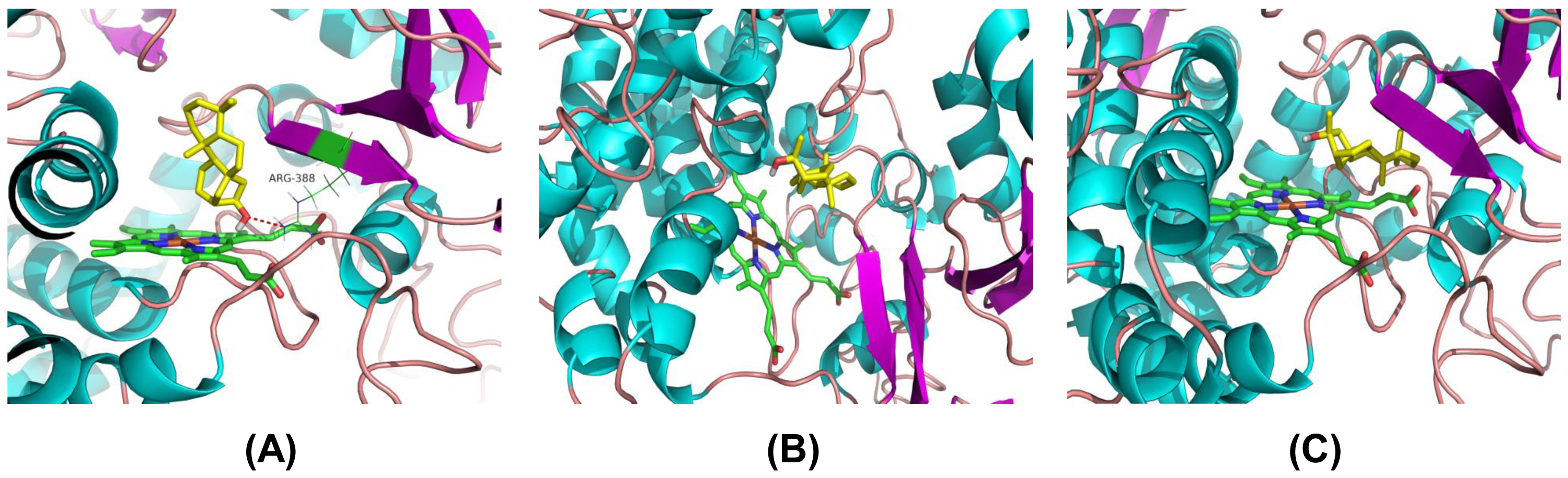


**Supplementary Figure S6. Binding modes of TwKO mutants L387D (A), L387R (B), L387S (C) with 16α-hydroxy*-ent*-kaurane (yellow) and haem (green carbon atoms).** The models were generated using 3DBG (structure of human *Streptomyces coelicolor* cytochrome P450 monooxygenase CYP170A1) as a template. The hydrogen bonding interactions are displayed in dotted lines.

**REFERENCES**

Davidson, S.E., Smith, J.J., Helliwell, C.A., Poole, A.T., and Reid, J.B. (2004). The pea gene LH encodes *ent*-kaurene oxidase. *Plant Physiol* 134**,** 1123-1134. doi: 10.1104/pp.103.032706.
